# Wing reduction influences male mating success but not female fitness in cockroaches

**DOI:** 10.1038/s41598-017-02647-7

**Published:** 2017-05-24

**Authors:** Michael Kotyk, Zuzana Varadínová

**Affiliations:** 1 Charles University, Faculty of Science, Department of Zoology, Prague, 12844 Czech Republic; 2 National Museum, Department of Zoology, Prague, 19300 Czech Republic

## Abstract

Although cockroaches (Blattodea s. str.) exhibit high proportion of species with reduced wings, the underlying evolutionary forces remain unclear. Wing reduction in insects is generally considered advantageous for females and a trade-off between investment into the flying apparatus and reproduction is predicted to explain its evolution. However, what if the wing maintenance is an important issue for males’ fitness? Males raise wings during the ritualized courtship which is viewed as an unavoidable movement unveiling the tergal glands for female access. We, however, propose a novel male mating success hypothesis suggesting that male wings are essential for their successful mating. We tested these two competing, but not mutually exclusive hypotheses in the cockroach *Eublaberus distanti*. We found no effect of female wing loss on any of the measured fecundity characteristics despite that alatectomized females histolyzed flight muscles. On the contrary, alatectomized males did not histolyze wing muscles, but experienced a markedly decreased mating success. Our findings, therefore, provide the first evidence on the crucial mechanical role of wings on male mating success. Consequently, selection for the retention of wings in males rather than for their reduction in females can explain the evolution of sexual wing dimorphism in cockroaches and other insects.

## Introduction

The development of wings and active flight is recognized as one of the most iconic evolutionary innovations in insects which not only enabled them to inhabit a plethora of habitats, but also drive the diversification to a countless number of forms^1, 2^. Nevertheless, a great number of representatives of almost all pterygote orders exhibit a certain degree of wing reduction^3–5^. Although it is not obvious why wing reduction and flight loss occurred so many times independently during insect evolution, recent studies surprisingly show that not only wing gain, but also wing loss is linked with a faster molecular evolution^6^ and acceleration of insect speciation^7^. Moreover, wing reduction is associated with numerous morphological, physiological, and behavioural changes which are of both ecological and evolutionary significance^8, 9^. Different evolutionary theories are predicted to cause the occurrence of reductive traits^10^. As for insect wings, it is widely accepted that they are frequently reduced in stable or isolated habitats where the dispersal of individuals is nearly impossible or not essential for their survival. Examples of such habitats include islands, caves, high mountains, or refugia^3^. According to the classical view, reductive traits in both sexes would then slowly appear due to accumulation of neutral mutations under relaxed selection^11^. However, the role of directional selection on evolution of reductive traits in such habitats should, at least in some cases, be considered as well^12^.

Nonetheless, in many insect taxa we’re dealing with sexual dimorphism in wings where one sex — almost exclusively females — exhibit higher degree of wing reduction than the other sex. In many cases, this dimorphism is so extensive that females are completely wingless, while males remain fully winged. In such instances, the prevailing assumption is that insect wing reduction is driven by directional selection. Fitness advantages such as extra energy, which can be invested into faster or higher reproduction of short-winged and wingless females in a resource-limited environment, may lead to the reduction of this expensive and dispensable trait. Indeed, such trade-off between investment into flying apparatus and reproduction (flight-oogenesis hypothesis) was repeatedly demonstrated in plentiful intrasexual wing dimorphic Orthoptera^13–15^, Hemiptera^16, 17^, or Lepidoptera^18, 19^. However, several studies do not provide support for this trade-off between energy costs of wing maintenance and reproduction^20–23^, suggesting that it is not the sole reason behind wing loss. Nevertheless, virtually all studies have focused on fitness of females, while little attention was paid to the fitness of males^24, 25^. Therefore, the question still remains, why do so many insect males retain fully developed wings whilst they are lost in females?

**Figure 1:**
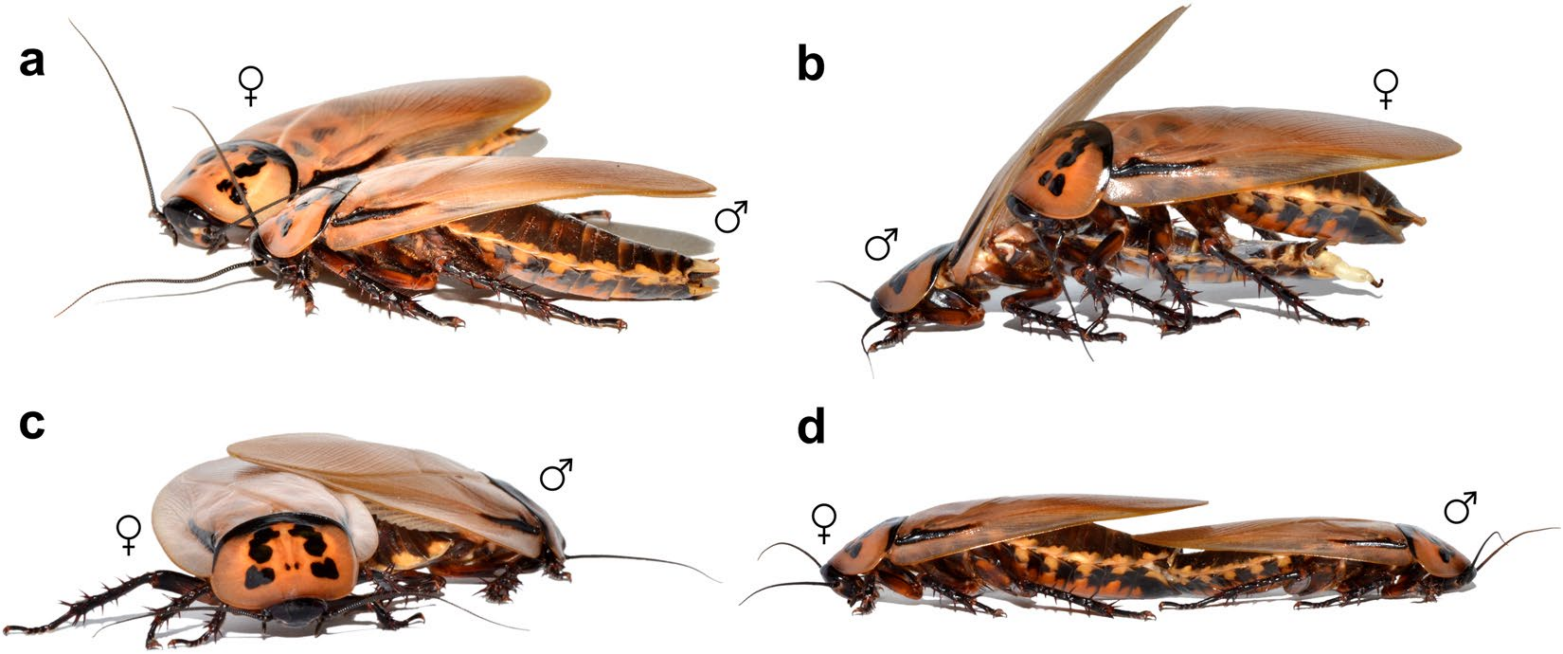
Ritualized courtship sequence as performed by *E. distanti*: (**a**) The male positions himself in front of the female, pumps his abdomen and raises his wings, (**b**) the female climbs on the male’s back where she is halted by his wings. The male then grasps the female genitalia by genital hook positioned on his right phallomere, (**c**) after genital connection is achieved, the male moves forward and the female performs a clockwise rotation 180 degrees off his back. Note the male wings pushing against the female body; (**d**) couple is then fixed in end-to-end position.

Cockroaches (Blattodea s. str., excluding termites), are one of the most diversified polyneopteran group containing more than 4500 species^26^ and exhibiting one of the highest occurrences of forms with reduced wings^3, 27–29^. They have achieved practically all possible wing conditions ranging from fully developed wings (macropterous), through forms with variable degree of reduction (brachypterous and micropterous), to those with completely absent wings (apterous) in one or both sexes. However, it is always the female which precedes the male in wing reduction leading to the frequently occurring sexual wing dimorphism. Despite the incredible diversity of cockroach wings, there is no study exploring the significance of their reduction and its evolutionary implications. Within the flight-oogenesis paradigm, one might explain the female wing reduction in cockroaches as a consequence of selection for higher or faster reproduction in those species where active flight is not inevitable for the survival. However, what if the wing maintenance, even in flightless form, is an important issue for males? We focused on the courtship behaviour which is highly ritualized and remarkably uniform among cockroaches^29^. Once in the vicinity of a receptive female, the male moves the abdomen up and down with the wings slightly raised and positions himself in front of the female (Fig. 1a). He then raises the wings higher to expose the whole abdominal dorsum. The female responds by climbing onto his back, where she is palpating the tergites and — if present — licking the products of tergal glands. In this moment, the male grasps the female genitalia by a genital hook positioned on the extensible phallomere (Fig. 1b). When connection is achieved, he moves forward, causing the female to rotate 180 degrees off his back (Fig. 1c). The pair is then stabilized in the typical end-to-end position (Fig. 1d) until the copulation is terminated. Some authors claim that palpating and licking the tergite glands is essential to maintain the female in a position allowing the connection^30, 31^. Furthermore, one would immediately expect that tergal glands may function as a nuptial gift. As a result, wing-raising, a distinct behavioural element performed during the courtship act, is simply viewed as an unavoidable movement which must be performed to unveil the tergal area for female access. However, it was shown that cockroach females did not gain any fitness benefit from copulating with a male with tergal secretions compared to males with tergal secretions experimentally removed^32^. Additionally, the male mating success and related behavioural parameters were not influenced by the absence of tergal secretions. Based on these facts and our preliminary observations on cockroach mating biology, we argue, it is rather the male wings that may help to manoeuvre the female into a position which enables the achievement of connection. Therefore, we propose a novel male mating success hypothesis which suggests that retention of wings in cockroach males is essential for their successful and timely mating. Consequently, males with reduced wings will be handicapped in mating compared to macropterous ones.

Thus, the main aim of our study was to test two competing, though mutually not exclusive hypotheses concerning wing reduction and loss in cockroaches: (i) flight-oogenesis hypothesis — females lose their wings to enhance their reproduction and (ii) male mating success hypothesis — males retain their wings to ensure their mating success. First, we evaluated the effect of wing loss on wing musculature histolysis. Then, we tested the influence of wing loss on female mating and reproduction success. Finally, we examined the influence of the male wing state on his mating success. To empirically test our goals, we chose South American macropterous cockroach *Eublaberus distanti* (Kirby, 1903) (Blaberidae: Blaberinae). Like the majority of members of the subfamily Blaberinae, they climb on walls and dig in organic debris of tree holes or caves and do not exhibit any willingness for active flight^33, 34^. Relocation of energy from wings and wing muscles is thus a possibility and most genera of Blaberinae (>70%, unpublished data) indeed reduce their wings in some way. Here we employ wing alatectomy to obtain brachypterous and apterous experimental groups from naturally macropterous individuals.

## Material and Methods

### Insects and rearing conditions

Four mass laboratory colonies of *E. distanti* cockroaches were used to obtain experimental individuals, i.e. adult virgin males and females of known age. Last-instar larvae were collected from the rearing colonies. Male and female larvae were identified and cockroaches of the same sex and origin (laboratory colony) were placed together into rearing boxes (160 × 160 × 110 mm). Larvae were checked daily for the occurrence of newly emerged adults (day 0), that were immediately transferred into small plastic boxes (165 × 108 × 65 mm) according to their age, sex, and origin. Only adults with no obvious external damage caused by improper or incomplete eclosion (e.g. non-overlapping or curled wings, old cuticle on the leg) were included in further experiments. In order to obtain individuals with partly (brachypterous group) and completely (apterous group) reduced wings, alatectomy was performed according to Roff^35^ on the day of final moult when flight muscles are not yet fully developed and functional^36, 37^. Brachypterous experimental group of cockroaches was created by cutting off wings transversely just above 2^nd^ abdominal tergite, while in apterous group wings were cut off at their base leaving ca 5 mm long stub of humeral angle. Laboratory colonies, experimental cockroaches in pre-experimental period and during all experiments were kept in the same room at 25 ± 2 °C, ambient r.h., and under L12:D12 photoperiod. Every box was supplied with food (dog pellets) and water (cotton wool plugged tube) ad libitum.

### Experiment 1

#### Can alatectomy lead to wing musculature histolysis

Adult males and females were split into two groups — experimental apterous group with wings cut off at the day of adult emergence and control macropterous group. After 70 days of keeping in rearing boxes, cockroaches were euthanatized by ethyl acetate. The dorsolongitudinal muscles (DVM) of mesothorax and methatorax were immediately dissected from each specimen and dried at 45 °C for 24 hours in a hybridization oven (Problot 12, Labnet). Cockroach bodies (without DVM) were dried at 70 °C for 24 hours in a Dry Air Sterilizer/Oven (FN120, Nüve). DVM and bodies (without DVM and wings) were weighted (KERN PFB 120-3) in fresh as well as dried state. All cockroaches used in the experiment were virgin. No clutch was produced during the life of females and no formed ootheca in a brood sac of females was observed during dissections. However, ovaries with developed, but green-coloured loose eggs were frequently detected.

### Experiment 2

#### Influence of wing loss on female reproduction success

Macropterous and apterous females at the age of 5 days after the last moult were individually introduced to three macropterous males. Mating tetrads were conducted in a glass arena (d = 300 mm Petri dish) and recorded with a static video camera (Sony Handycam HDR-SR11E 60 GB HD Camcoder, Japan) positioned above the arena for analysis of the influence of female wing condition on her mating performance. If the female successfully mated with one of the males within 30 minutes, the constituted pair was transferred into the plastic breeding box (165 × 108 × 65 mm). Each female was kept in the presence of the mated male for their whole life in order to avoid the stress from social isolation^38, 39^. Moreover, single mating of different quality males can provide enough sperm for different number of clutches, which is, according to our unpublished findings, always lower than the females’ lifetime clutch production. On the contrary, multiple mating, which we indeed repeatedly observed during the experiment, can reduce the influence of current male condition on female reproduction output. Males are however generally not as long-lived as females. In case of the early male death, he was replaced by a female larva as a social companion. The pairs were checked for the newborn clutches or aborted ootheca every day until the natural death of females. We recorded the following parameters related to female reproduction fitness: number of clutches (total number of oothecae produced during lifetime), number of fertile clutches (oothecae from which living larvae hatched), number of sterile clutches (oothecae with no viable larva), time to produce the first clutch (in days), viable larvae per clutch (number of viable larvae hatched per fertile ootheca), and total female fecundity (number of larvae produced during the female’s lifetime). We further checked the female weight (weight at the age of 5 days, measured before mating) and female longevity (time from adult emergence to death) which presumably influence female reproduction too.

### Experiment 3

#### The role of male wings on male mating success

Macropterous, brachypterous, or apterous male at the age of 5 days after the last moult was individually introduced to virgin macropterous female of the same age. Mating dyads were conducted in a glass arena (d = 300 mm Petri dish) and recorded with a static video camera (Sony Handycam HDR-SR11E 60 GB HD Camcoder, Japan) positioned above the arena for further analysis of mating sequences. The following behavioural acts were quantified: latency of courtship (time from the first male contact with female until the start of male courtship display), female courtship answer (latency from the beginning of male courtship to females’ first climb on the males’ back), courtship duration (time from the beginning of courtship until the fixation of connected pair into the end-to-end position), number of climbs (how many times the female climbed on the males’ back until the genitalia connection was achieved), mating success (frequency of successful matings) and finally mating duration (time from the pair fixation into the end-to-end position until the pair decoupling). If the mating did not occur within 30 minutes, the experiment was terminated as unsuccessful. To compare total female fecundity, females accompanied by female larvae were kept in breeding boxes until their natural death.

### Data analyses

Video records of mating sequences were checked in GOM player 2.2 (Gretech Corporation, South Korea). All statistical analyses and graph constructions were performed in R 3.0.2 (R Foundation for Statistical Computing, Vienna, Austria). All characteristics analyzed in Exp. 1 had normal distribution and were compared by Welch Two Sample t-test. Statistical tests applied in Exp. 2 consisted of ANOVA (males size comparison), Pearson Chi-square test (influence of male size on mating performance), Welsch t-test (comparison of female reproduction characteristics with normal distribution), Asymptotic Wilcoxon Mann-Whitney Rank Sum Test (comparison of mating and reproduction characteristics without normal distribution), and generalized linear models with Poisson distribution and link function log (comparison of no. of climbs on males back). In Exp. 3, we used Pearson Chi-square test to evaluate the influence of male wing state on mating success. Latency of the beginning of courtship from the first encounter and female courtship answer were suffering with considerable overdispersion. Thus, generalized linear models with quasipoisson distribution were used to compare these parameters. Other characteristics (no. of climbs, courtship duration) with Poisson distribution did not suffer by overdispersion, so generalized linear models with Poisson distribution and link function log were used. Mating duration and total female fecundity were compared by one-way ANOVA. All data obtained and analyzed in this study are available in Supplementary ﻿﻿Information.

## Results

### Experiment 1

#### Can alatectomy lead to wing musculature histolysis

There was no difference (t_12.378_ = 1.7497, p = 0.1049) between dry body weight (without wings) of macropterous (1757.27 ± 42.27 mg, n = 15) and apterous (1898.75 ± 68.93 mg, n = 8) females. We can thus presume there was no negative effect of alatectomy on the physiological condition of females. Contrary to that, significant difference in dry DVM weight between macropterous (48.87 ± 2.04 mg) and apterous (38.00 ± 1.90 mg) female group was revealed (t_19.441_ = −3.8900, p = 0.0009). The relative dry DVM weight (dry DVM weight/dry body weight) was also different (t_20.035_ = −4.8059, p = 0.0001) between macropterous (2.73 ± 0.14%) and apterous females (1.96 ± 0.08%). This indicates that alatectomy in cockroach females is accompanied by histolysis of wing muscles. As for males, we also found no difference (t_24.581_ = −0.1834, p = 0.856) between dry body weight (without wings) of macropterous (1123.50 ± 70.09, n = 14) and apterous (1083.92 ± 71.91, n = 13) group. Negative effect of alatectomy on their physiological condition can be rejected too. However, we also found no difference (t_24.893_ = −0.3941, p = 0.6969) in dry DVM weight between macropterous (36.92 ± 2.84 mg) and apterous (36.15 ± 3.12 mg) males. The relative dry DVM weight was comparably higher than in females but did not differ (t_24.977_ = −0.0375, p = 0.973) between macropterous (3.30 ± 0.26%) and apterous males (3.29 ± 0.29%).

### Experiment 2

#### Influence of wing loss on female reproduction

We conducted 70 successful mating trials with three macropterous males offered to individual macropterous or apterous female. Males within the trial differed in their size (F_2,207_ = 46.84, p < 0,0001); each female thus had a choice among big-sized (3.51 ± 0.05 g), mid-sized (3.23 ± 0.04 g), and small-sized (2.92 ± 0.05 g) male. Nevertheless, males courted and females mated randomly. The male size did not affect the first male who courted the female (χ^2^ = 3.9714, p = 0.1373), the first male climbed by a female (χ^2^ = 2.0857, p = 0.3524), nor the male’s mating success (χ2 = 4.9143, p = 0.0857). We consider it unsurprising in an experimental design where males did not have the chance to establish dominance hierarchies. Females who were in their receptive period did not exhibit any choosiness for the mate. In 70% of the trials they immediately mated with the first male that exhibited courtship behaviour towards her. Macropterous and apterous females did not show any difference in number of climbs (z = −1.145, p = 0.252) or in mating duration (z = −1.2519, p = 0.2106) indicating that female wing condition did not have any influence on the courtship and mating performance.

To compare reproductive fitness of macropterous and apterous females, we decided to exclude 15 observations from statistical analyses due to any of the following reasons: (i) the female’s partner died before the first clutch was born thus making repeated mating and fulfilling the female’s theoretical lifetime clutch production unfeasible; (ii) the female had never produced any viable larvae which could be caused by infertility of either male or female; (iii) the female died before the first clutch was produced. Our results, surprisingly, revealed no effect of alatectomy on females’ fecundity in any measured reproductive characteristic. There was no difference in the total number of clutches (z = −0.6826, p = 0.4949) as well as in the number of fertile (z = −0.7863, p = 0.4423) or sterile (z = −0.5062, p = 0.6127) clutches. Macropterous and apterous females did not differ in time to produce the first clutch (z = −1.606, p = 0.1083). Moreover, we did not find any difference in the number of viable larvae per clutch (t_53_ = 0.1071, p = 0.9151) or total female fecundity (t_53_ = −0.559, p = 0.5789). Macropterous and apterous female groups did not differ in their weight (t_53_ = −1.6429, p = 0.1067). Although a statistically significant difference in the female longevity was found (z = −2.1497, p = 0.0316), with macropterous females living 47 days longer than apterous group, we argue it has no or minimal impact on the addressed issues because: (i) total female fecundity was not dependent on the longevity (F_24 = _1.183, p = 0.2875); (ii) 47 days is less than the shortest interval between two successive clutches we recorded (that is 60 days); (iii) longevity of apterous females (308 ± 14.72 days) was long enough to produce 4 clutches (300.19 ± 6.10 days), which is the number of clutches during which female exploits most of their reproductive potential. Only 7 macropterous and 7 apterous females survived until the production of the fifth ootheca, of which 4 and 6, respectively, were sterile. No female produced more than 5 oothecae with viable nymphs. Table 1 summarizes the values of females’ condition and fecundity variables between macropterous and apterous group.

**Table 1:** Comparison of reproductive and condition parameters between macropterous and apterous females in Experiment 2:

	macropterous females		apterous females		test	p
	N	mean ± se	N	mean ± se		
number of clutches	26	3.96 ± 0.20	29	3.89 ± 0.28	Z = −0.6826	0.4949
number of fertile clutches	26	3.19 ± 0.18	29	3.00 ± 0.15	Z = −0.7683	0.4423
number of sterile clutches	26	0.77 ± 0.17	29	0.89 ± 0.25	Z = −0.5062	0.6127
time to produce the first clutch (days)^a^	25	79.28 ± 2.86	28	73.71 ± 2.19	Z = −1.606	0.1083
viable larvae per clutch	26	26.00 ± 1.72	29	26.26 ± 1.66	t_53_ = 0.1071	0.9151
total female fecundity^b^	26	83.88 ± 8.14	29	78.31 ± 5.76	t_53_ = −0.559	0.5789
female weight (g)	26	4.71 ± 0.09	29	4.52 ± 0.08	t_53_ = −1.6429	0.1067
female longevity (days)	26	355.88 ± 15.12	29	308.79 ± 14.32	Z = −2.1497	0.0315

^a^Observation was excluded if the first produced clutch was sterile, while the second and following ones were fertile. ^b^Number of larvae produced during the female’s lifetime.

### Experiment 3

#### The function of male wings in mating

We conducted 62 mating dyads with receptive virgin female randomly offered to one of macropterous (n = 22), brachypterous (n = 22) or apterous (n = 18) male. We did not find any difference in latency of courtship across the three groups (GLM, F_2.82_ = 0.3876, p = 0.6799). Regardless of the male wing condition, the courtship display performed was identical and included all the behavioural acts as described in the introduction. Even the apterous males were painstakingly raising their wing stubs despite the absence of wings itself. When looking at the occurrence of the female courtship answer, male wing condition did not have any effect on latency from the beginning of male courtship to females’ first climb on the males’ back (GLM, F_2.81_ = 0.4256, p = 0.6548); it took 15.13 ± 3.08 s in trials with macropterous, 19.97 ± 4.50 s in trials with brachypterous and 18.16 ± 2.98 s in trials with apterous male. However, what the wing state definitely does affect is the correct execution of the whole courtship sequence and achievement of successful mating. According to our observations, the wings play an essential role in two aspects of the courtship ritual. Firstly, they are necessary to halt the female on the male back in such position, that the male is able to grasp female’s genitalia with his phallus (Fig. 1b). Secondly, the overlap of male wings over the female body is required to fix the horizontal plane of connection during the rotation of the female (Fig. 1c). If the wings are shortened or absent, the female cannot stop in the right place on the male’s back or the rotation is performed at a wrong angle and the couple disconnects easily.

**Figure 2:**
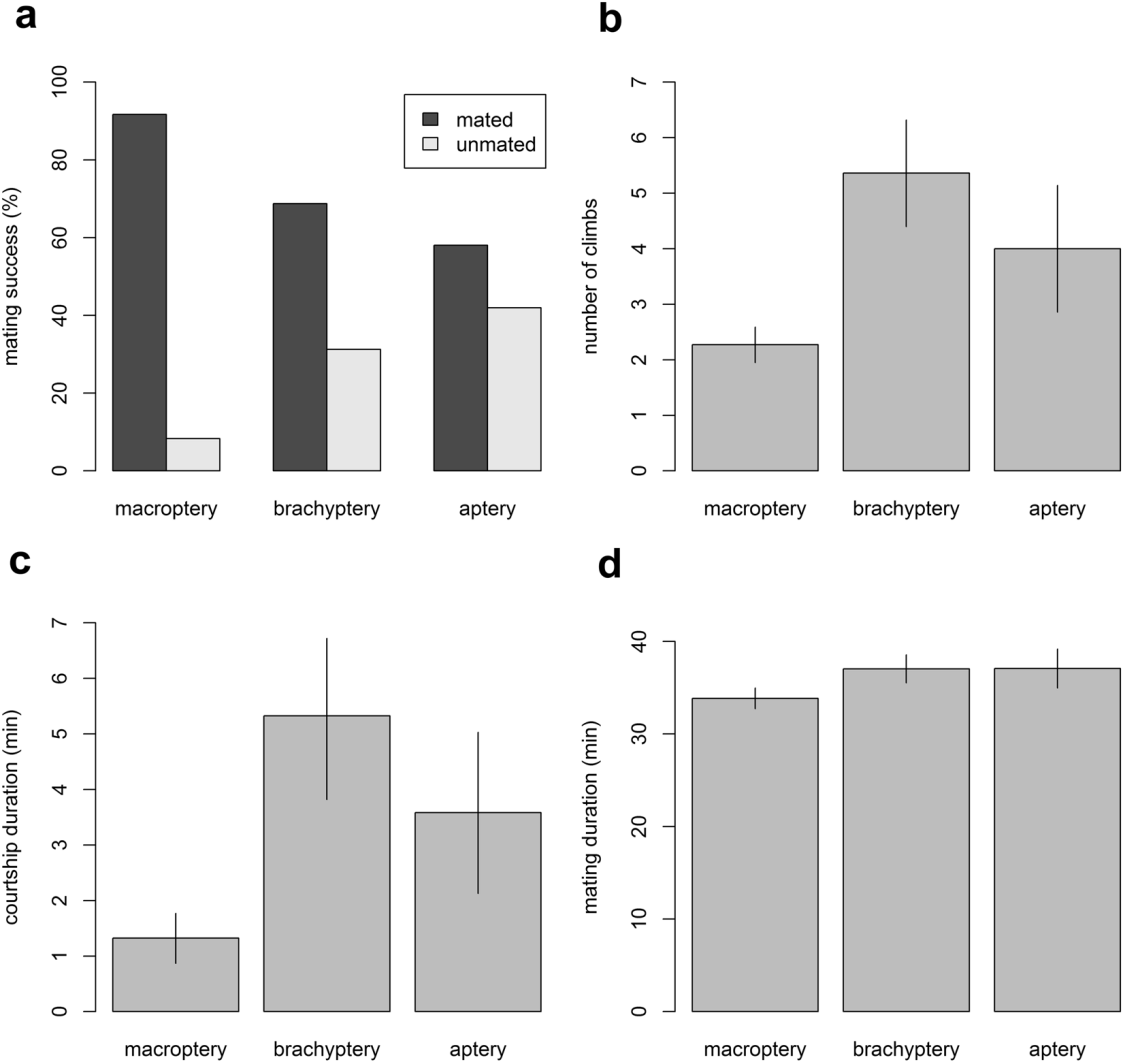
(**a**) Mating success of males based on their wing treatment (macroptery, brachyptery, aptery) in Experiment 3. The males which successfully mated across the three groups were then compared for (**b**) number of climbs, (**c**) courtship duration and (**d**) mating duration. Whiskers are SE.

In terms of numbers, partial or complete absence of wings decreased the mating success of males (χ^2^_2_ = 7.6148, p = 0.0222). While 91.8% of macropterous males successfully mated, only 68.8% of brachypterous and 58% of apterous males achieved mating (Fig. 2a). Comparing only the males which successfully mated across the three groups, wing reduction significantly increased the number of climbs on the male abdomen (p < 0.0001, Fig. 2b) as well as the courtship duration (p < 0.0001, Fig. 2c). It took 2.27 ± 0.32 climbs and 1.32 ± 0.45 minutes to achieve mating from the beginning of courtship when the male was macropterous. By contrast, 5.36 ± 0.96 climbs and 5.27 ± 1.45 minutes were needed in the case of brachypterous and 4.00 ± 1.14 climbs and 3.58 ± 1.45 minutes in the case of apterous males. After successful mating is achieved, wing state does not affect the mating duration (F_2,59_ = 1.429, p = 0.248, Fig. 2d). Additionally, wing state of the male who mated had no significant impact on the total female fecundity (F_2.54_ = 0.744, p = 0.48, counted for females living longer than 60 days).

## Discussion

We firstly determined whether alatectomy leads to wing muscle histolysis in cockroaches. Consistent with methodologically similar work on crickets^35^, we have shown that DVM dry weight of apterous females was significantly lower than in macropterous ones. Our results indicate that wing loss in female cockroaches is accompanied by histolysis of wing muscles and the released energy may be utilized elsewhere. Contrary to that, we have not found such an effect in the case of males. It can be assumed that cockroach females, as the sex much more burdened with offspring production, are more ready and capable to take advantage of released resource reserves such as those stored in flight musculature. Besides, macropterous males possess markedly bigger relative DVM weight than macropterous females. We suppose this is because male wings, unlike those in females, are still actively used in other than flying context. It is during the courtship ritual when males raise their wings. Even apterous males were diligently raising their wing stubs when facing the receptive female in Experiment 3. The objection, that males in our first experiment were kept separately from females to prevent mating, and thus did not perform wing raising, could be raised. However, same-sex sexual behaviour is well known among cockroach males and other insects^40, 41^. In our male experimental group, we indeed repeatedly observed male displaying the courtship ritual to another male, occasionally leading to short male-male connection. Taking into account all these biological aspects, it is not very surprising that, as opposed to females, males possess relatively bigger wing muscles and do not histolyse them.

In Experiment 2, we examined the trade-off between investment into the flying apparatus and reproduction (flight-oogenesis hypothesis). Our results are in sharp contrast with most of the previous studies^13–19^. Surprisingly, none of the reproductive characteristic compared in the present study differed between macropterous and apterous females. The absence of differences in reproduction-related traits might be explained in several ways. One explanation is that cockroaches are not preadapted for wing loss triggered fecundity enhancement. They are thus unable to enhance their offspring production even though they are capable of histolysing their flight muscles. The second possibility is that it is not the reproduction enhancement but another not measured fitness-related characteristic which is associated with female wing loss. The third plausible explanation is that females have already reduced their wing muscles into a physiological minimum necessary to carry unused wings and allocated the energy into reproduction. Energy release associated with definite wing loss is then too low to further enhance reproduction. Lower relative DVM weight of females compared to males from Experiment 1 would comply with this explanation. We suppose that at least in Blaberinae cockroaches, wing state has no effect on female reproduction and the reason for the occurrence of wing reduction might lay elsewhere. However, as there are no comparable data available for other cockroaches, we cannot definitely exclude that our observations are specific for this case only.

In Experiment 3, macropterous, brachypterous, or apterous individual males were exposed to receptive female to investigate the influence of wing condition on male mating success. We provide strong evidence that shortened or absent wings lowered the mating success and increased the courtship duration and the number of climbs. According to our observations male wings play a key role in two aspects of the courtship ritual. Firstly, they act as a mechanical stop which halts the female on the male’s back in such a position that the male can easily grasp the female’s genitalia with his phallus. It is at this point where partly brachypterous, but especially apterous males were handicapped. In such cases, female cannot stop at the right place on the male’s back and often just continues climbing until she overpasses the male’s head. Secondly, male wings overlapping the female body are required to fix the horizontal plane of connection during the rotation of the female. At this point, both apterous and brachypterous males are handicapped in the same way. When wings are shortened or absent the rotation is often performed at a wrong angle and the couple disconnects directly during the rotation or a few seconds after establishing end-to-end position. If male wings indeed play such a crucial function, a more dramatic decrease in mating success might be expected for brachypterous and apterous males. However, in our experimental design females were exposed to a single male. Consequently, they did not have any other option and sooner or later accepted the male who was at hand. Whereas in natural conditions Blaberinae cockroaches are gregarious^29, 34, 42^ and many males are present at once. Any unsuccessful mating attempt or extension of courtship duration may thus immediately lead to replacement of the unsuccessful male by another male and to the ultimate failure of an individual’s reproduction success.

Since phylogenetic approach has revealed trait reductions are widespread and can even be much more common than trait gains, reductive traits have become an intensively studied phenomenon^43^. Among numerous examples, understanding insect wing reduction and occurrence of wing dimorphism has gained a lot of attention^8, 9, 17, 44–46^. Most studies dealing with fitness advantages connected to sexual dimorphism in wings are, however, focused on female fitness gains of the reduction. Contrary to that, only few previous studies tested potential male fitness gain or constrains of reduction^24, 25^. Our study thus brings unique results in three aspects: (i) it is the first study experimentally exploring fitness advantages and constrains of wing reduction in cockroaches; (ii) we did not find any relationship between female wing reduction and enhanced reproduction; (iii) we propose and tested an alternative explanation that wings are inevitable for male fitness. In the light of our results, we infer it is the male mating success hypothesis which might explain the frequent occurrence of sexual wing dimorphism in cockroaches. This hypothesis suggests that, because wings are essential for successful and timely mating of cockroach males, there is a strong selection for retention of male wings. On the other hand, frequent wing reductions in cockroach females are probably not shaped by the trade-off between flight and reproduction. A plausible explanation could be an accumulation of neutral mutations under relaxed selection, although this remains to be tested.

Adaptive traits can become non-functional and free for decay as a result of environmental shifts or changes in life-history characteristics^10, 11, 47^. Hence, we suppose that male wing reduction in cockroaches is very probably correlated with a change of courtship and mating behaviour. It is surely not a coincidence that Madagascar hissing cockroaches, with both sexes apterous, exhibit a mating pattern in which the female neither climbs on the male’s back nor performs any rotation. The connection is achieved by the male simply moving backwards and grasping the female genitalia^48^. As for brachypterous males, there is a whole range from slightly reduced to almost completely reduced wings, but we tested only males with wings shortened to 2^nd^ abdominal tergite. Whether variable degrees of wing reduction decrease the male mating success differently, remains to be investigated. Our hypothesis thus calls for further studies assessing the evolutionary relationship between wing states and mating diversity in a group of related species of cockroaches.

It is questionable if our hypothesis could help us understand the evolution of sexual wing dimorphism in other insect groups. However, in some cases, male retention of wings coadapted for novel, most probably mating related function, seems to be conceivable. Interestingly, a very similar example can be found in katydids and grasshoppers where male wings are used for production of signal calls. Some Tettigoniidae have lost their wings, in that females are apterous, yet males retain short forewings that function only in stridulation^49^. When studying evolution of wing reduction, it is important to keep in mind that wings do not exhaust their purpose solely with flight. After all, it is highly probable that both insect^50^ and bird^51^ wings did not evolve for the purpose of flight in the first place.

## Electronic supplementary material

Supplementary information
