# Supplementary material for: Wing reduction influences male mating success but not female fitness in cockroaches
Source: Sci Rep. 2017 May 24;7:2367. doi: 10.1038/s41598-017-02647-7 (PMC5443839; doi:10.1038/s41598-017-02647-7)
Supplement: Supplementary file 1 — Supplementary information [file 41598_2017_2647_MOESM1_ESM.pdf]

Flying on the wings of love: wing state in cockroaches influence male mating success but not female fitness

Michael Kotyk[1] and Zuzana Varadínová[1,2,*]

[1]Charles University, Faculty of Science, Department of Zoology, Prague, 12844, Czech Republic

[2]National Museum, Department of Zoology, Prague, 19300, Czech Republic

*Author for correspondence: Z.V. (varadino@natur.cuni.cz)

Experiment 1 - Can alatectomy lead to wing musculature histolysis? – DISSECTION DATA

cockroach ID	sex	wings	fresh body weight (g)	fresh body without wing weight (g)	fresh DVM (g)	dry body weight (g)	dry body weight without wings (g)	dry DVM (g)	dry mass wing/dry body weight (%)
1	female	macroptery	3.996	3.868	0.165	1.592	1.496	0.051	3.297
5	female	macroptery	4.268	4.147	0.217	1.895	1.788	0.066	3.560
6	female	macroptery	4.774	4.665	0.173	2.074	1.922	0.054	2.733
7	female	macroptery	4.272	4.17	0.217	1.825	1.687	0.060	3.434
9	female	macroptery	3.905	3.784	0.178	1.579	1.499	0.056	3.601
10	female	macroptery	4.381	4.241	0.129	1.777	1.688	0.042	2.428
11	female	macroptery	4.346	4.207	0.158	2.028	1.938	0.049	2.466
12	female	macroptery	4.251	4.11	0.107	1.825	1.742	0.040	2.245
13	female	aptery	4.249	4.249	0.094	1.601	1.601	0.031	1.900
14	female	aptery	4.790	4.79	0.134	1.971	1.971	0.040	1.989
15	female	aptery	4.681	4.681	0.150	2.162	2.162	0.045	2.039
16	female	aptery	4.653	4.653	0.120	2.070	2.070	0.038	1.803
17	female	aptery	4.579	4.579	0.128	1.883	1.883	0.038	1.978
18	female	aptery	3.869	3.869	0.124	1.633	1.633	0.036	2.157
19	female	aptery	4.561	4.561	0.106	1.942	1.942	0.031	1.571
20	female	aptery	4.655	4.655	0.148	1.928	1.928	0.045	2.281
21	female	macroptery	4.910	4.748	0.136	2.150	2.055	0.048	2.282
22	female	macroptery	4.103	3.957	0.139	1.707	1.627	0.054	3.212
23	female	macroptery	4.373	4.229	0.110	1.888	1.807	0.039	2.113
24	female	macroptery	4.147	4.003	0.132	1.678	1.596	0.045	2.742
25	male	macroptery	3.199	3.104	0.207	1.088	1.013	0.059	5.504
25	female	macroptery	4.457	4.31	0.130	1.830	1.741	0.045	2.520
26	male	aptery	2.633	2.633	0.200	0.865	0.865	0.055	5.978
26	female	macroptery	4.587	4.442	0.124	2.001	1.916	0.042	2.145
27	male	aptery	3.628	3.628	0.175	1.437	1.437	0.046	3.102
27	female	macroptery	4.546	4.408	0.132	1.948	1.857	0.042	2.212
28	male	aptery	3.325	3.325	0.216	1.390	1.390	0.053	3.673

29	male	macroptery	3.068	2.97	0.183	1.169	1.070	0.052	4.635
30	male	macroptery	3.208	3.11	0.168	1.285	1.197	0.045	3.623
31	male	macroptery	3.22	3.14	0.080	1.444	1.38	0.024	1.709
32	male	macroptery	3.184	3.055	0.129	1.32	1.238	0.040	3.130
33	male	macroptery	3.989	3.854	0.135	1.629	1.548	0.049	3.068
34	male	macroptery	4.081	3.962	0.119	1.742	1.576	0.030	1.868
35	male	macroptery	2.626	2.513	0.113	0.971	0.899	0.032	3.437
36	male	aptery	1.798	1.724	0.074	0.721	0.721	0.025	3.351
37	male	aptery	2.869	2.764	0.105	0.883	0.883	0.032	3.497
38	male	aptery	3.29	3.164	0.126	1.417	1.417	0.038	2.612
39	male	aptery	2.873	2.756	0.117	1.108	1.108	0.046	3.986
40	male	aptery	3.149	3.037	0.112	1.315	1.315	0.037	2.737
41	male	aptery	2.809	2.702	0.107	0.988	0.988	0.035	3.421
42	male	aptery	2.653	2.576	0.077	0.941	0.941	0.024	2.487
43	male	aptery	3.054	2.954	0.100	1.244	1.244	0.034	2.660
44	male	aptery	2.689	2.626	0.063	1.083	1.083	0.020	1.813
45	male	macroptery	2.83	2.755	0.075	1.193	1.114	0.024	2.109
46	male	macroptery	2.854	2.757	0.097	1.195	1.135	0.033	2.825
47	male	macroptery	2.443	2.336	0.107	1.094	1.034	0.036	3.364
48	male	macroptery	1.911	1.825	0.086	0.769	0.707	0.030	4.071
49	male	aptery	2.109	2.054	0.055	0.699	0.699	0.025	3.453
50	male	macroptery	2.565	2.465	0.100	1.188	1.116	0.034	2.957
51	male	macroptery	1.898	1.814	0.084	0.728	0.702	0.029	3.967

Experiment 2 - Influence of wing loss on female reproduction success – MATING DATA

Female ID	F wing	F weight (g)	Male big weight	Male mid weight (g)	Male small weight (g)	first male courted	first male climbed	mated male	mated M weight (g)	number of climbs	mating duration (s)	notes on bad observations
2	macroptery	4.71	3.83	3.4	2.87	1	1	1	3.83	1	171	outlier
3	macroptery	4.95	4.14	3.67	3.43	2	1	2	3.67	2	2797	
6	macroptery	6.23	4.11	3.74	3.62	1	1	1	4.11	1	2720	
7	macroptery	5.36	3.66	3.23	2.73	3	1	1	3.66	7	2875	
9	macroptery	5.26	3.87	3.62	3.26	1	1	3	3.26	3	2354	
10	macroptery	5.45	3.46	3.18	3.04	1	1	1	3.46	1	2354	
12	macroptery	4.73	2.98	2.95	2.74	1	1	1	2.94	1	1603	
14	aptery	3.983	3.89	3.48	3.19	3	2	2	3.48	3	3442	
15	macroptery	4.05	3.13	3.12	2.99	2	2	1	3.13	3	1670	
17	macroptery	4.48	3.85	3.28	2.83	3	3	3	2.83	1	2020	
18	macroptery	5.36	3.46	3.37	3.09	1	1	3	3.09	4	2065	
19	aptery	5.005	3.55	3.24	3.08	1	1	1	3.55	1	1930	
20	macroptery	4.51	3.55	3.43	3.35	1	2	3	3.35	8	2483	
22	aptery	4.603	3.66	3.57	3.53	2	2	1	3.66	1	1920	
26	macroptery	4.040	3.49	3.39	3.15	3	3	3	3.15	1	3009	
27	aptery	4.248	3.45	3.16	2.98	1	1	1	3.45	1	1935	
28	aptery	4.619	3.19	3.03	2.94	3	3	3	2.94	1	1904	
29	aptery	4.181	3.21	3.16	3.16	1	1	1	3.21	1	1770	
30	aptery	4.481	3.49	3.31	2.79	1	1	1	3.49	7	120	outlier
31	macroptery	3.820	3.42	3.09	2.94	3	1	3	2.94	2	1870	
33	aptery	4.907	3.56	3.27	3.07	1	1	1	3.56	1	1834	
34	aptery	4.288	3.19	2.98	2.86	2	2	3	2.86	5	1560	
38	macroptery	5.080	3.15	2.75	2.57	1	1	1	3.15	1	1342	
39	macroptery	4.700	3.03	2.99	2.76	2	3	2	2.99	7	1345	
41	macroptery	5.170	3.16	3.07	2.92	3	2	3	2.92	2	1590	
42	macroptery	4.410	3.32	2.9	2.12	2	2	2	2.9	1	1584	
44	aptery	4.405	3.74	3.44	3.27	2	2	1	3.74	3	1342	
45	aptery	4.307	3.71	3.12	2.67	3	2	2	3.12	11	1655	alarm

												behaviour of the group
46	aptery	4.519	3.72	3.4	3.37	2	2	2	3.4	1	2188	
47	aptery	4.187	4.09	3.82	3.14	3	3	3	3.14	1	2210	
48	macroptery	4.020	3.79	3.76	3.54	1	1	1	3.79	1	1702	
49	aptery	3.973	4.14	3.57	3.49	3	2	3	3.49	5	2580	
50	macroptery	4.570	3.31	3.26	2.92	1	3	3	2.92	1	1526	
51	aptery	4.172	3.55	3.44	2.95	1	3	3	2.95	1	1324	
55	aptery	4.671	3.58	3.29	3.02	1	1	1	3.58	7	3261	
56	aptery	4.854	3.82	3.28	2.38	1	1	1	3.82	5	1717	
59	aptery	4.210	2.86	2.32	2.17	3	3	3	2.17	1	2277	
60	aptery	3.968	3.45	3.4	3.29	3	3	3	3.29	1	1800	
61	aptery	5.224	2.87	2.65	2.47	1	2	2	2.65	1	2263	
62	macroptery	4.070	3.28	3.21	3.19	1	1	1	3.28	1	1950	
73	aptery	4.365	3.069	2.981	2.865	3	3	2	2.981	2	1410	
74	aptery	5.135	4.092	3.376	3.049	2	2	2	3.376	1	1629	
75	aptery	4.922	3.566	3.384	3.324	2	2	2	3.384	1	1831	
76	macroptery	4.924	3.453	2.953	2.348	3	3	2	2.953	6	1805	
77	aptery	4.400	3.746	3.455	2.471	1	3	1	3.746	2	2078	
78	macroptery	4.218	3.711	2.891	2.329	3	3	3	2.329	1	1750	
79	macroptery	4.945	3.619	3.349	3.116	2	2	2	3.349	1	1804	
80	aptery	4.895	3.947	3.368	2.833	3	3	3	2.833	3	1702	
81	aptery	4.272	4.707	3.711	3.499	1	1	1	4.707	2	1626	
82	aptery	4.498	4.088	4.063	3.184	3	3	3	3.184	1	1604	
94	aptery	4.525	3.207	2.652	1.91	2	1	1	3.207	1	1463	
95	aptery	4.628	3.383	3.265	2.176	1	3	1	3.265	5	1486	
96	macroptery	4.644	2.964	2.96	2.254	1	1	1	2.964	1	1917	
101	aptery	4.578	2.832	2.679	2.601	3	3	3	2.601	6	2062	
105	macroptery	4.287	3.396	3.166	2.422	1	1	1	3.396	1	1680	
106	macroptery	4.704	3.035	2.93	2.537	1	1	1	3.035	1	1988	
108	aptery	4.754	3.308	3.218	2.515	2	2	2	3.218	1	1766	
109	macroptery	5.182	3.778	3.422	3.353	1	1	1	3.778	1	1832	
116	aptery	5.301	3.276	3.098	2.952	3	3	2	3.098	2	3270	

129	macroptery	5.069	3.654	3.639	3.098	1	1	1	3.654	2	2108	
130	macroptery	4.88	4.28	2.863	2.775	2	2	2	2.863	4	2726	
164	aptery	5.449	3.507	3.337	3.076	2	2	1	3.507	13	2910	alarm behaviour of the group
168	aptery	4.049	3.168	3.12	3.003	2	3	2	3.12	11	1780	
169	aptery	4.381	3.456	3.337	3.205	3	3	3	3.205	1	1585	
170	aptery	4.197	2.895	2.759	2.603	1	1	1	2.895	1	1578	
175	aptery	4.243	3.742	3.542	3.422	3	3	1	3.742	4	1124	
176	aptery	3.672	3.622	3.175	3.166	2	2	2	3.175	2	1782	
177	aptery	4.155	3.024	3.018	2.947	1	1	1	3.024	10	1182	
186	aptery	NA	3.58	3.305	2.93	1	1	1	3.58	2	1590	
187	aptery	5.56	2.916	2.733	2.423	2	2	2	2.733	35	1490	alarm behaviour of the group

Experiment 2 - Influence of wing loss on female reproduction success – REPRODUCTION DATA

Female ID	F Wing	F weight (g)	courted M weight (g)	F longevity (days)	N clutches	N fertile clutches	N sterile clutches	total F fecundity	time to the first clutch (days)
2	macroptery	4.710	3.83	318	3	3	0	97	63
3	macroptery	4.950	3.67	392	5	5	0	167	65
6	macroptery	6.230	4.11	435	6	5	1	181	58
9	macroptery	5.260	3.26	366	5	4	1	92	75
10	macroptery	5.450	3.46	357	5	4	1	142	68
12	macroptery	4.730	2.94	390	3	2	1	45	77
14	aptery	3.983	3.48	380	3	2	1	24	NA
15	macroptery	4.050	3.13	423	4	3	1	61	77
17	macroptery	4.480	2.83	480	6	4	2	76	74
18	macroptery	5.360	3.09	439	5	4	1	97	70
19	aptery	5.005	3.55	408	6	3	3	112	91
20	macroptery	4.510	3.35	325	4	4	0	115	66
22	aptery	4.603	3.66	276	3	3	0	82	61
26	macroptery	4.040	3.15	418	4	4	0	76	71
28	aptery	4.619	2.94	321	4	4	0	79	70
29	aptery	4.181	3.21	312	3	3	0	30	63
30	aptery	4.481	3.49	292	4	2	2	42	98
33	aptery	4.907	3.56	432	4	4	0	122	64
34	aptery	4.288	2.86	494	5	5	0	106	64
38	macroptery	5.080	3.15	391	3	2	1	70	114
39	macroptery	4.700	2.99	303	4	3	1	114	97
41	macroptery	5.170	2.92	408	4	4	0	139	68
42	macroptery	4.410	2.9	260	3	2	1	30	74
44	aptery	4.405	3.74	266	4	3	1	23	64
45	aptery	4.307	3.12	307	4	4	0	103	60
46	aptery	4.519	3.4	249	3	3	0	97	66
47	aptery	4.187	3.14	241	3	3	0	37	64
48	macroptery	4.020	3.79	362	3	3	0	32	75

49	aptery	3.973	3.49	249	3	3	0	88	65
50	macroptery	4.570	2.92	272	3	3	0	33	75
55	aptery	4.671	3.58	267	4	3	1	90	61
59	aptery	4.210	2.17	254	3	2	1	27	85
60	aptery	3.968	3.29	276	3	3	0	52	75
61	aptery	5.224	2.65	331	4	4	0	119	64
62	macroptery	4.070	3.28	250	3	3	0	65	77
73	aptery	4.365	2.981	402	8	3	5	80	71
74	aptery	5.135	3.376	396	6	3	3	99	74
75	aptery	4.922	3.384	257	2	2	0	65	84
76	macroptery	4.924	2.953	207	3	2	1	53	83
77	aptery	4.400	3.746	229	2	2	0	54	92
78	macroptery	4.218	2.329	366	4	4	0	56	86
79	macroptery	4.945	3.349	396	3	2	1	61	NA
80	aptery	4.895	2.833	259	3	3	0	92	84
82	aptery	4.498	3.184	393	6	4	2	111	82
95	aptery	4.628	3.265	266	3	3	0	76	85
96	macroptery	4.644	2.964	384	4	3	1	50	97
101	aptery	4.578	2.601	204	2	2	0	70	77
105	macroptery	4.287	3.396	181	3	2	1	63	78
108	aptery	4.754	3.218	223	3	2	1	74	95
109	macroptery	5.182	3.778	380	4	3	1	92	109
129	macroptery	5.069	3.654	464	6	2	4	47	101
130	macroptery	4.88	2.863	286	3	3	0	127	84
164	aptery	5.449	3.507	184	3	2	1	85	65
170	aptery	4.197	2.895	406	7	3	4	107	65
176	aptery	3.672	3.175	381	5	4	1	125	75

Experiment 3 - The role of male wings on male mating success – MATING DATA

Male ID	M wing	mating success	latency contact-courtship (s)	latency courtship-first climb (s)	courtship duration (s)	number of climbs	mating duration (s)
1	macroptery	1	10	4	25	2	1894
2	aptery	0	1	9	NA	11	NA
3	macroptery	1	19	11	65	6	1506
4	macroptery	1	3	7	135	4	1486
5	aptery	1	1	53	57	1	1551
6	macroptery	1	40	56	59	1	1673
7	aptery	1	0	13	18	1	1223
8	macroptery	1	6	9	11	1	1956
14	macroptery	1	7	7	15	2	1648
15	aptery	0	4	8	NA	14	NA
16	aptery	0	60	41	NA	19	NA
18	brachyptery	0	3	73	NA	17	NA
19	brachyptery	1	14	4	6	1	1615
20	aptery	1	3	22	318	6	1428
21	brachyptery	0	4	9	NA	16	NA
29	macroptery	1	2	8	134	4	2453
36	brachyptery	1	2	418	1642	4	1248
40	brachyptery	1	70	70	76	1	2077
51	macroptery	1	2	24	29	1	2029
53	macroptery	0	8	16	NA	6	NA
59	macroptery	1	1	9	73	3	1977
62	macroptery	1	1	14	23	1	1962
63	macroptery	1	4	6	17	1	1790
64	aptery	1	15	45	1413	18	2620
65	aptery	1	2	3	551	9	2579
71	macroptery	0	5	5	NA	7	NA
72	brachyptery	0	0	8	NA	22	NA
73	brachyptery	0	0	1	NA	19	NA

74	brachyptery	1	3	29	1025	10	2284
75	aptery	1	0	6	20	2	2166
76	macroptery	1	57	53	74	2	1990
79	aptery	0	0	29	NA	11	NA
80	brachyptery	1	57	117	124	1	1993
81	brachyptery	1	0	10	316	10	2076
82	aptery	1	2	12	22	2	2065
84	brachyptery	1	14	2	35	2	1857
85	aptery	1	12	21	23	1	1952
86	aptery	1	1	44	53	1	2477
87	brachyptery	1	1	65	69	1	2503
89	aptery	1	12	11	40	3	2136
90	brachyptery	1	4	24	31	1	2038
91	macroptery	1	89	18	22	1	2170
92	aptery	0	3	5	NA	23	NA
93	brachyptery	1	2	2	75	5	2361
94	macroptery	1	2	6	8	1	1962
96	brachyptery	1	11	13	414	11	2053
97	brachyptery	1	182	6	22	1	2280
98	macroptery	1	4	5	12	1	1930
99	brachyptery	1	15	10	539	12	2053
101	macroptery	1	3	3	21	2	2068
102	macroptery	1	NA	NA	NA	4	2650
103	brachyptery	0	47	87	NA	14	NA
104	brachyptery	0	4	3	NA	11	NA
105	aptery	0	13	22	NA	7	NA
106	brachyptery	1	1	2	156	6	2950
107	aptery	1	4	64	79	2	2074
108	macroptery	1	5	5	170	4	2130
109	brachyptery	1	5	11	742	13	2314
110	brachyptery	1	5	16	420	8	2300
111	brachyptery	1	5	22	429	9	2381

112	brachyptery	0	13	40	NA	9	NA
114	aptery	0	19	3	NA	10	NA
115	aptery	0	53	15	NA	12	NA
116	aptery	1	55	11	771	6	3604
117	brachyptery	1	9	52	129	4	3185
118	macroptery	1	31	32	159	4	2121
119	aptery	0	30	4	NA	15	NA
120	brachyptery	1	2	2	11	1	2959
121	aptery	1	2	3	32	2	2421
122	brachyptery	0	3	3	NA	11	NA
123	macroptery	1	4	3	8	1	2613
124	aptery	1	10	39	44	1	2697
125	aptery	0	10	5	NA	2	NA
127	macroptery	1	23	21	577	3	2390
128	aptery	1	11	2	6	1	2143
129	aptery	1	4	18	22	1	2209
130	brachyptery	1	6	1	3	1	1957
131	brachyptery	0	4	5	NA	20	NA
132	brachyptery	1	9	7	32	3	2239
133	aptery	0	3	7	NA	16	NA
134	macroptery	1	13	26	30	1	2278
135	aptery	0	6	8	NA	25	NA
136	aptery	1	2	9	345	13	2588
137	brachyptery	1	2	5	665	13	2163
138	aptery	1	5	8	55	2	2099
139	brachyptery	0	12	17	NA	16	NA
140	aptery	0	4	23	NA	22	NA

Experiment 3 - The role of male wings on male mating success – MATED MALES DATA

Male ID	M wing	M weight (g)	F weight (g)	Number of climbs	Mating duration (s)	F longevity (days)	total female fecundity
1	macroptery	3.120	4.990	2	1894	375	0
3	macroptery	3.790	4.300	6	1506	NA	2
4	macroptery	3.260	4.360	4	1486	439	12
5	aptery	3.120	4.703	1	1551	NA	19
6	macroptery	3.630	4.660	1	1673	NA	13
7	aptery	3.832	5.19	1	1223	257	42
8	macroptery	4.210	4.420	1	1980	352	10
14	macroptery	3.059	5.008	2	1648	257	12
19	brachyptery	4.066	4.494	1	1615	379	0
20	aptery	3.652	4.516	6	1428	231	0
29	macroptery	3.878	4.913	4	2453	407	34
36	brachyptery	3.576	4.464	4	1248	342	42
40	brachyptery	4.174	4.93	1	2077	212	46
51	macroptery	3.888	4.935	1	2029	240	64
59	macroptery	3.164	4.155	3	1977	222	0
62	macroptery	3.245	3.766	1	1962	40	0
63	macroptery	3.025	4.020	1	1790	111	11
64	aptery	3.206	4	18	2620	254	57
65	aptery	2.698	4.069	9	2579	234	0
74	brachyptery	3.722	4.983	10	2284	337	83
75	aptery	3.495	5.319	2	2166	371	0
76	macroptery	4.005	5.027	2	1990	31	0
80	brachyptery	4.142	4.458	1	1993	287	2
81	brachyptery	3.941	4.25	10	2076	167	32
82	aptery	3.836	4.731	2	2065	183	38
84	brachyptery	4.144	5.018	2	1857	315	65
85	aptery	3.854	4.874	1	1952	401	52
86	aptery	3.166	4.725	1	2477	NA	37
87	brachyptery	3.544	4.605	1	2503	120	16

89	aptery	3.950	5.438	3	2136	211	23
90	brachyptery	3.799	4.874	1	2038	413	58
91	macroptery	4.131	4.568	1	2170	147	27
93	brachyptery	3.998	5.399	5	2361	29	0
94	macroptery	4.361	4.777	1	1962	384	25
96	brachyptery	4.541	5.3	11	2053	397	31
97	brachyptery	3.510	4.49	1	2280	308	0
98	macroptery	4.052	4.551	1	1930	391	113
99	brachyptery	3.822	5.043	12	2053	178	42
101	macroptery	3.853	5.383	2	2068	378	98
106	brachyptery	4.427	5.347	6	2950	440	47
107	aptery	3.961	4.148	2	2074	486	11
108	macroptery	4.396	5.649	4	2130	128	0
109	brachyptery	5.020	5.268	13	2314	99	18
110	brachyptery	3.571	5.046	8	2300	238	63
111	brachyptery	3.895	5.365	9	2381	325	78
116	aptery	3.842	5.684	6	3604	35	0
117	brachyptery	4.721	4.572	4	3185	321	41
118	macroptery	3.800	5.016	4	2121	168	20
120	brachyptery	4.119	6.023	1	2959	262	58
121	aptery	4.340	4.232	2	2421	422	0
123	macroptery	3.630	5.942	1	2613	480	39
124	aptery	3.970	5.57	1	2697	143	28
127	macroptery	4.431	4.956	3	2390	315	61
128	aptery	4.226	5.028	1	2143	420	50
129	aptery	3.949	5.22	1	2209	405	75
130	brachyptery	4.273	6.296	1	1957	495	77
132	brachyptery	5.343	6.184	3	2239	444	0
134	macroptery	4.405	5.176	1	2278	57	0
136	aptery	4.111	5.517	13	2588	454	16
137	brachyptery	3.870	5.154	13	2163	443	49
138	aptery	4.074	5.155	2	2099	378	97
